# Post-endoscopic neurosurgical *Candida albicans* meningoencephalitis diagnosed by targeted next-generation sequencing: a case report

**DOI:** 10.3389/fmed.2026.1747908

**Published:** 2026-05-07

**Authors:** Luwen Huang, Yalin Xi, Ming Yu, Linlin Li, Ping Chen

**Affiliations:** 1 Department of Neurology, Suining Central Hospital, Suining, China; 2 Department of Pharmacy, Suining Central Hospital, Suining, China

**Keywords:** *Candida albicans*, cerebrospinal fluid, infection, meningoencephalitis, targeted next-generation sequencing

## Abstract

*Candida albicans* (*C. albicans*) meningoencephalitis is a rare but diagnostically challenging infection that usually occurs in immunocompromised individuals, although life-threatening disease can also develop in immunocompetent patients. The absence of specific clinical features and the limited sensitivity of conventional microbiological methods frequently lead to diagnostic delays. We describe a 79-year-old woman with no known immunodeficiency who presented with symptoms initially suggestive of bacterial meningitis and failed to improve after empirical ceftriaxone, meropenem, and vancomycin therapy, while repeated blood and cerebrospinal fluid (CSF) cultures remained negative. Her history of endoscopic transsphenoidal surgery raised concern for an atypical postoperative infection. Targeted next-generation sequencing (tNGS) of the CSF subsequently provided important etiological evidence for *C. albicans* and supported timely initiation of amphotericin B deoxycholate and flucytosine, followed by oral fluconazole, resulting in marked clinical and biochemical improvement. This case highlights the diagnostic difficulty of intracranial Candida infection. In this patient, CSF tNGS provided important etiological evidence when routine microbiological tests remained negative and contributed to timely targeted antifungal treatment.

## Introduction

*Candida albicans* is the most frequently reported pathogenic *Candida* species (1, 2). Although CNS infection caused by *Candida* is uncommon, delayed recognition may lead to inappropriate treatment and an unfavorable outcome (3–5). Diagnosis is particularly difficult after neurosurgical procedures because the clinical presentation, cerebrospinal fluid (CSF) abnormalities, and imaging findings are often nonspecific and may overlap with those of bacterial or tuberculous meningitis (6, 7). When routine microbiological tests remain negative, timely pathogen identification becomes especially important, and targeted next-generation sequencing (tNGS) may provide useful evidence to support the diagnosis (8, 9).

## Case report

On January 27, 2025, a 79-year-old woman was admitted to the Department of Neurology at Suining Central Hospital with complaints of headache and decreased responsiveness. She had a history of cardiac pacemaker implantation 1 year prior and no known history of hypertension, diabetes, or immunodeficiency. She denied any history of drug or food allergies, recent travel, or contact with animals. Two months earlier, she had undergone endoscopic transnasal resection of a pituitary adenoma at another hospital, followed by a repeat endoscopic transnasal evacuation of a sellar hematoma due to postoperative bleeding.

On admission, her vital signs were as follows: temperature 37.4 °C, pulse rate 88 beats/min, respiratory rate 20 breaths/min, blood pressure 131/83 mmHg, and heart rate 88 beats/min. Lung auscultation was clear, with no rales detected. Cardiac rhythm was regular. Neurological examination revealed that the patient was conscious and articulate but exhibited slowed responses. The pupils were equal in size (3 mm) with sluggish direct and consensual light reflexes. Bilateral nasolabial folds were symmetrical, and the tongue was midline. Muscle strength was grade 5, muscle tone was normal, and tendon reflexes were brisk (++). The Romberg test could not be performed because of poor cooperation, and meningeal signs were negative.

**Figure 1:**
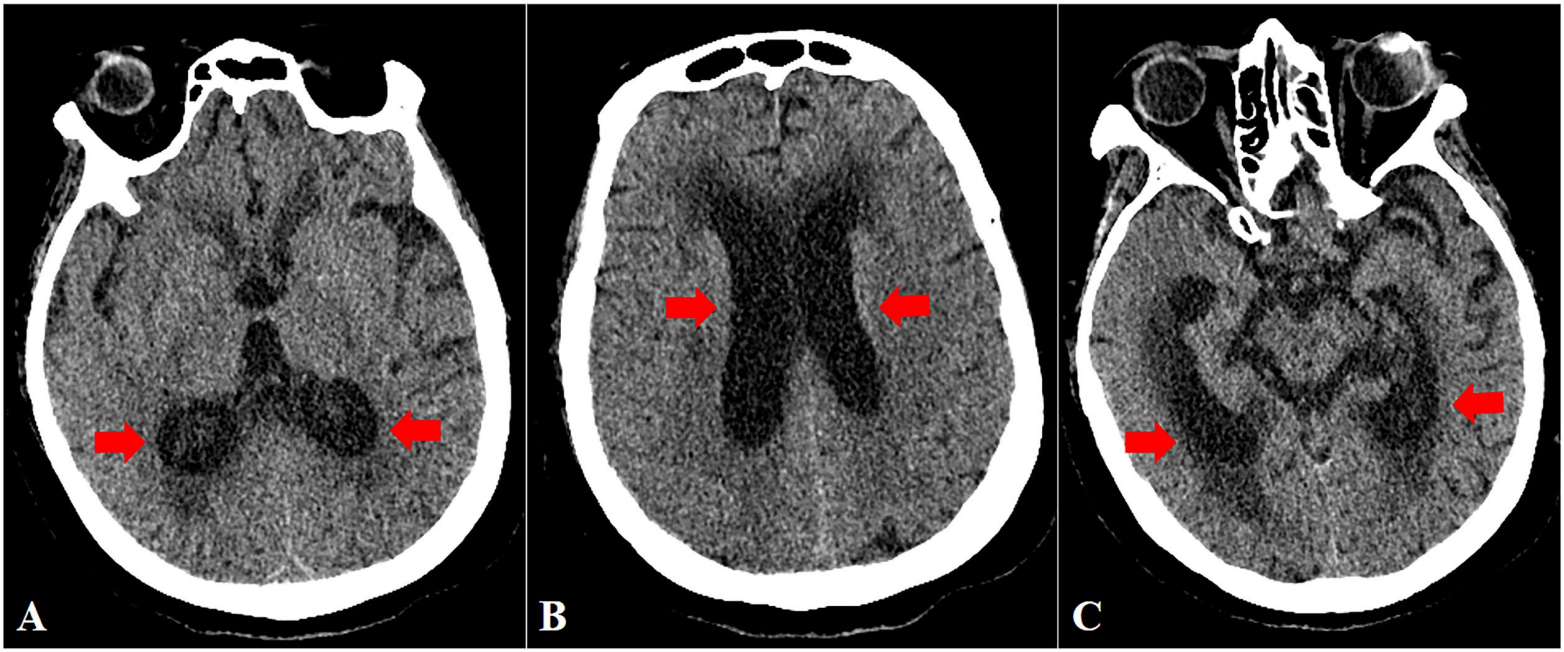
Cranial CT demonstrates bilateral ventricular hydrocephalus. Red arrows indicate dilation of the lateral ventricles.

Laboratory tests revealed a white blood cell (WBC) count of 10.31 × 10^9^/L (reference range: 3.5–9.5 × 10^9^/L), neutrophil count of 6.74 × 10^9^/L (1.8–6.3 × 10^9^/L), C-reactive protein (CRP) level of 17.86 mg/L (<10 mg/L), and serum albumin level of 36.58 g/L. Human immunodeficiency virus (HIV) testing and serologic testing for syphilis were both negative. Brain computed tomography (CT) demonstrated mild ventriculomegaly with slight hydrocephalus (Figure 1). Magnetic resonance imaging (MRI) was not performed because of the implanted cardiac pacemaker. Chest CT findings were unremarkable.

After admission, the patient’s body temperature increased to 38.1 °C. Lumbar puncture revealed an opening pressure of 220 mmH₂O (normal: 80–180 mmH₂O). The CSF was clear and colorless. CSF analysis showed a WBC count of 804 × 10^6^/L (normal: 0–8 × 10^6^/L), glucose level of 0.87 mmol/L (2.5–4.5 mmol/L), and protein level of 1.28 g/L (0.15–0.45 g/L). Microbiological tests of the CSF, including Gram staining, India ink staining, acid-fast staining, *Mycobacterium tuberculosis* DNA testing, and quantitative detection of herpes simplex virus type 1 (HSV-1) and HSV-2 DNA, were all negative.

A multidisciplinary team review was conducted, and the differential diagnosis included bacterial meningitis, tuberculous meningitis, viral encephalitis, fungal meningitis, and postoperative chemical meningitis. Tuberculous meningitis was considered because the CSF showed low glucose and elevated protein. However, it was considered less likely because the patient had no symptoms suggestive of tuberculous intoxication, such as low-grade fever, night sweats, or anorexia, no cranial nerve abnormalities such as ptosis, diplopia, or facial weakness, and negative acid-fast staining and *Mycobacterium tuberculosis* DNA results. Viral encephalitis was also considered less likely because the markedly reduced CSF glucose and elevated protein were atypical for a viral etiology, and both HSV-1 and HSV-2 DNA tests were negative. Fungal infection was not strongly suspected at the initial stage because the patient had no known history of immunodeficiency, HIV testing was negative, and there was no history of steroid exposure or prolonged antibiotic exposure. In addition, the India ink test was negative, and postoperative bacterial meningitis was considered more common in the early clinical setting. Postoperative chemical meningitis was also considered, but it was regarded as less likely because the patient had persistent fever and marked inflammatory changes in the CSF. Based on the overall clinical presentation, postoperative bacterial meningitis was initially regarded as the most probable diagnosis.

Empirical antibacterial therapy with ceftriaxone at 2.0 g every 12 h was therefore initiated on hospital day 2. However, the patient remained febrile, with a peak temperature of 39 °C, and showed no clinical improvement. Blood cultures obtained on day 4 were negative. Meropenem at 2.0 g every 8 h and vancomycin at 0.5 g every 6 h were therefore added empirically. After 4 days of combined antibacterial therapy, the patient remained febrile, and her level of consciousness deteriorated to a light coma. Subsequent fungal biomarker assays showed an elevated serum 1,3-*β*-D-glucan level of 120 pg./mL, whereas the galactomannan test was negative. In view of the poor response to broad-spectrum antibacterial therapy and repeatedly negative conventional microbiological studies, tNGS was subsequently performed for further etiological evaluation of the central nervous system infection (Supplementary File 1).

**Figure 2:**
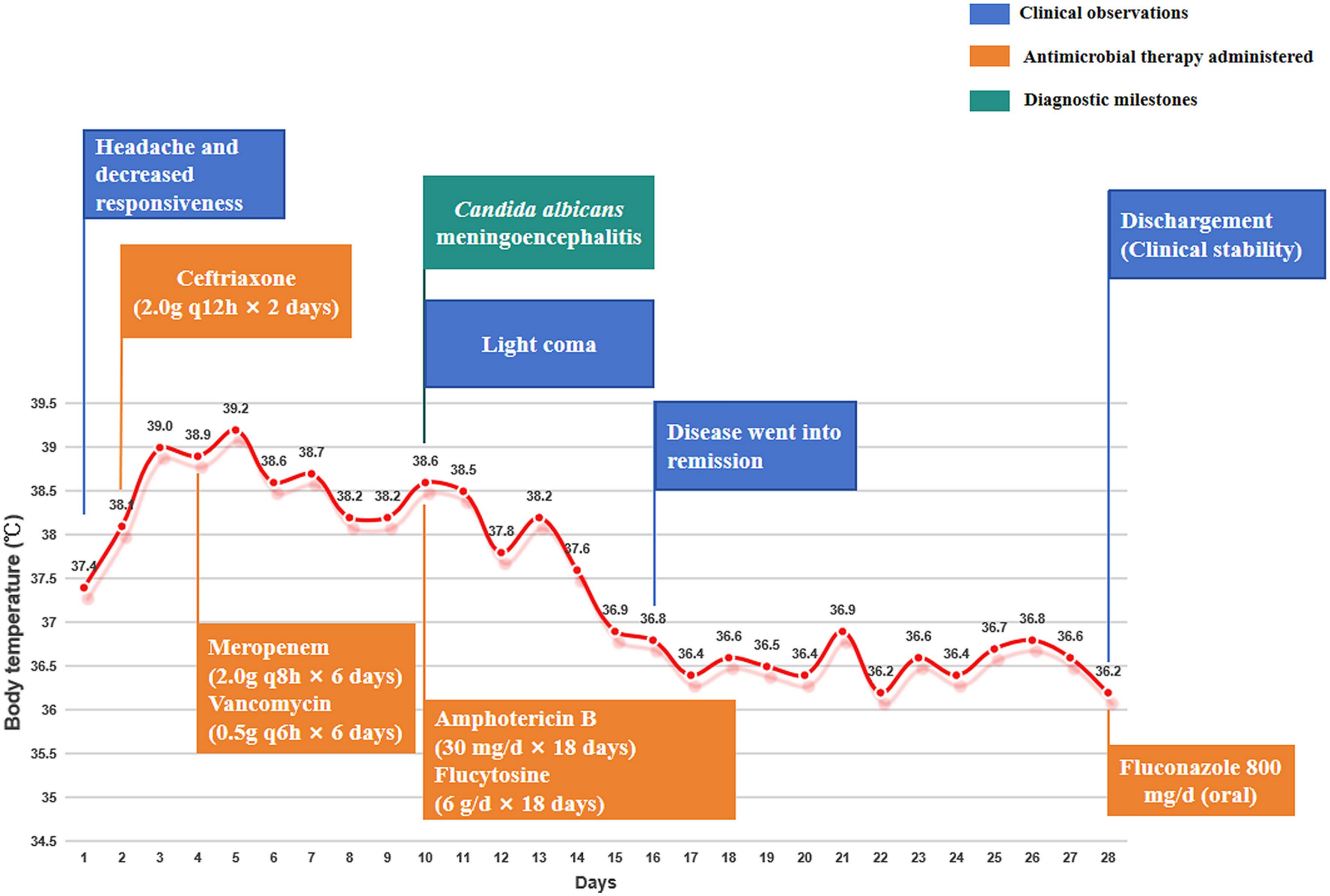
Integrated timeline of the patient’s clinical course during hospitalization.

CSF tNGS was subsequently performed using a commercial multiplex targeted amplification and high-throughput sequencing assay. The report identified *C. albicans* with a normalized sequence count of 57, which was defined by the laboratory as the number of microbial sequences per 100,000 raw reads. The report also indicated that assay quality control was acceptable, including total sequence count, Q30 ratio, internal control, positive control, and negative control (Supplementary File 2). Considering the recent neurosurgical history, CSF abnormalities, repeatedly negative conventional microbiological studies, poor response to broad-spectrum antibacterial therapy, the CSF tNGS finding, and the subsequent response to antifungal treatment, a final diagnosis of *Candida albicans* meningoencephalitis was established. On hospital day 9, antimicrobial therapy was switched to amphotericin B deoxycholate (initially at 5 mg/day and gradually increased to 30 mg/day) in combination with flucytosine (1.5 g four times daily). Following this regimen change, the patient showed marked clinical improvement. By hospital day 16, her level of consciousness had improved from shallow coma to somnolence, with noticeably better responsiveness. CSF biochemical and cytological parameters also improved significantly (Table 1). By hospital day 28, her slowed responsiveness had nearly resolved, and her body temperature had returned to normal (Figure 2). After 20 days of antifungal therapy, however, the patient and her family declined further inpatient treatment and follow-up brain CT because of financial constraints, and she was discharged on oral fluconazole at 800 mg/day as maintenance therapy. At a two-month outpatient follow-up, the patient remained clinically stable with no evidence of recurrence. The timeline of antimicrobial therapy is illustrated in Figure 2.

**Table 1:** Patient’s CSF test results.

Category	Laboratory tests	Normal range	Results			
			Day 1	Day 8	Day 16	Day 25
Appearance and pressure	Appearance	Clear and transparent	Light yellow slightly turbid	Light yellow slightly turbid	Light yellow slightly turbid	Clear and transparent
	Pressure (mmH_2_O)	80 ~ 180	320*	350*	220*	165
Inflammatory markers	WBC (cells/μL)	0 ~ 5	804*	580*	270*	60*
	Protein quantification (g/L)	0.15 ~ 0.45	1.28*	2.01*	1.32*	0.86*
Metabolic values	Chloride (mmol/L)	120 ~ 132	123	116*	121	122
	Glucose (mmol/L)	2.4 ~ 4.5	0.87*	1.7*	2.5	2.6

*Indicates abnormal values.

## Discussion

This case highlights the diagnostic challenge of postoperative *Candida albicans* central nervous system infection. At the initial stage, fungal CNS infection was not strongly suspected because the patient presented with postoperative fever, headache, and markedly abnormal cerebrospinal fluid findings, which were initially more suggestive of postoperative bacterial meningitis. In addition, the clinical manifestations, CSF abnormalities, and cranial CT findings were nonspecific and did not clearly distinguish fungal infection from bacterial or tuberculous meningitis. MRI could not be performed because of the implanted cardiac pacemaker, which further limited early etiological assessment. However, the patient showed a poor response to broad-spectrum antibacterial therapy, while conventional microbiological studies remained repeatedly negative. These findings raised suspicion of an alternative etiology and prompted further pathogen-directed investigation.

The postoperative setting was likely an important predisposing factor in this case. Although the pathogenesis of fungal CNS infection has not been fully elucidated, penetration across the blood–brain barrier (BBB) is considered the most likely mechanism in *Candida* meningitis (2, 10). Pathogens may cross the BBB through transcellular migration, paracellular migration, or the Trojan horse mechanism (2, 10). Because this patient had undergone two endoscopic neurosurgical procedures 2 months earlier, we speculate that recent surgical disruption of the BBB, together with postoperative factors and possible device-related colonization, may have contributed to the development of CNS infection.

Another key feature of this case was the difficulty of early etiological diagnosis. Conventional microbiological methods, including blood culture, CSF staining, and routine pathogen testing, remained repeatedly negative (8, 9). Although culture remains the gold standard for diagnosing candidiasis, it may be insensitive and time-consuming, which can delay appropriate antifungal treatment (8, 9). In this case, CSF tNGS provided important etiological evidence supporting *C. albicans* meningoencephalitis when conventional microbiological tests remained repeatedly negative. Recent comparative studies suggest that targeted NGS may perform similarly to, or in some CSF settings better than, metagenomic NGS (mNGS), although these findings appear to depend on the specimen type and clinical context (11, 12). Therefore, in this case, tNGS should be regarded as a useful adjunctive diagnostic tool, but its result should still be interpreted together with the overall clinical and laboratory findings.

The therapeutic response in this case was also clinically informative. Amphotericin B combined with flucytosine remains the recommended first-line therapy for *C. albicans* meningitis, whereas fluconazole and voriconazole are considered alternative options (8). Echinocandins such as caspofungin, micafungin, and anidulafungin achieve CSF concentrations of only 1 to 5% of their plasma levels and are therefore not recommended as monotherapy for CNS infections. Treatment duration should be individualized according to the patient’s clinical condition, although the optimal duration remains uncertain (13). In this case, amphotericin B deoxycholate combined with flucytosine led to marked improvement in both clinical symptoms and laboratory parameters. After 20 days of antifungal therapy, the patient was discharged at her own request and transitioned to oral fluconazole for maintenance treatment. During follow-up, she remained clinically stable without evidence of recurrence, although repeat CSF and hematologic evaluations were not performed because of financial constraints.

This case provides several practical clinical lessons. First, postoperative fungal CNS infection should remain in the differential diagnosis when patients fail to respond to broad-spectrum antibacterial therapy and conventional microbiological studies remain negative. Second, nonspecific symptoms, CSF changes, and neuroimaging findings may delay recognition of fungal infection. Third, tNGS may provide useful etiological support in challenging cases, especially when routine diagnostic methods are inconclusive.

This case has several limitations. First, long-term functional and cognitive outcomes could not be fully assessed because no formal post-discharge cognitive evaluation was available. Second, a comprehensive immunological assessment was not performed. Although HIV testing was negative and there was no history of steroid exposure or prolonged antibiotic exposure, CD4/CD8 lymphocyte counts and serum immunoglobulin levels were not assessed. Therefore, the patient could only be described as having no known immunodeficiency. Third, the commercial tNGS report did not provide the assay positivity threshold, absolute total read count, or detailed background contamination data, and repeat tNGS was not performed. Finally, follow-up was incomplete because the patient declined repeat CSF testing and follow-up imaging, so treatment response was assessed mainly by clinical improvement rather than microbiological or radiological confirmation of cure.

## Conclusion

This case highlights that patients who develop persistent headache and fever after endoscopic neurosurgery should be evaluated for possible *Candida albicans* central nervous system infection, especially when they respond poorly to conventional antibacterial therapy. In this setting, CSF tNGS may serve as a useful adjunctive etiological tool when routine microbiological studies are inconclusive, although the results should be interpreted together with the clinical and laboratory context. Timely recognition of the infection and prompt initiation of targeted antifungal therapy were associated with clinical improvement in this patient. Amphotericin B combined with flucytosine remains the first line regimen for Candida meningitis.

## Data Availability

The original contributions presented in the study are included in the article/Supplementary material, further inquiries can be directed to the corresponding authors.

## Supplementary material

SUPPLEMENTARY FILE 1 Cerebrospinal fluid targeted next-generation sequencing (tNGS) results demonstrating the detection of Candida albicans (Chinese Version).

SUPPLEMENTARY FILE 2 Cerebrospinal fluid targeted next-generation sequencing (tNGS) results demonstrating the detection of Candida albicans (English Version).
