# Supplementary material for: Post-endoscopic neurosurgical Candida albicans meningoencephalitis diagnosed by targeted next-generation sequencing: a case report
Source: Front Med (Lausanne). 2026 May 7;13:1747908. doi: 10.3389/fmed.2026.1747908 (PMC13189900; doi:10.3389/fmed.2026.1747908)
Supplement: Supplementary file 1 [file Data_Sheet_1.pdf]

中枢神经系统多种病原体靶向
测序报告单

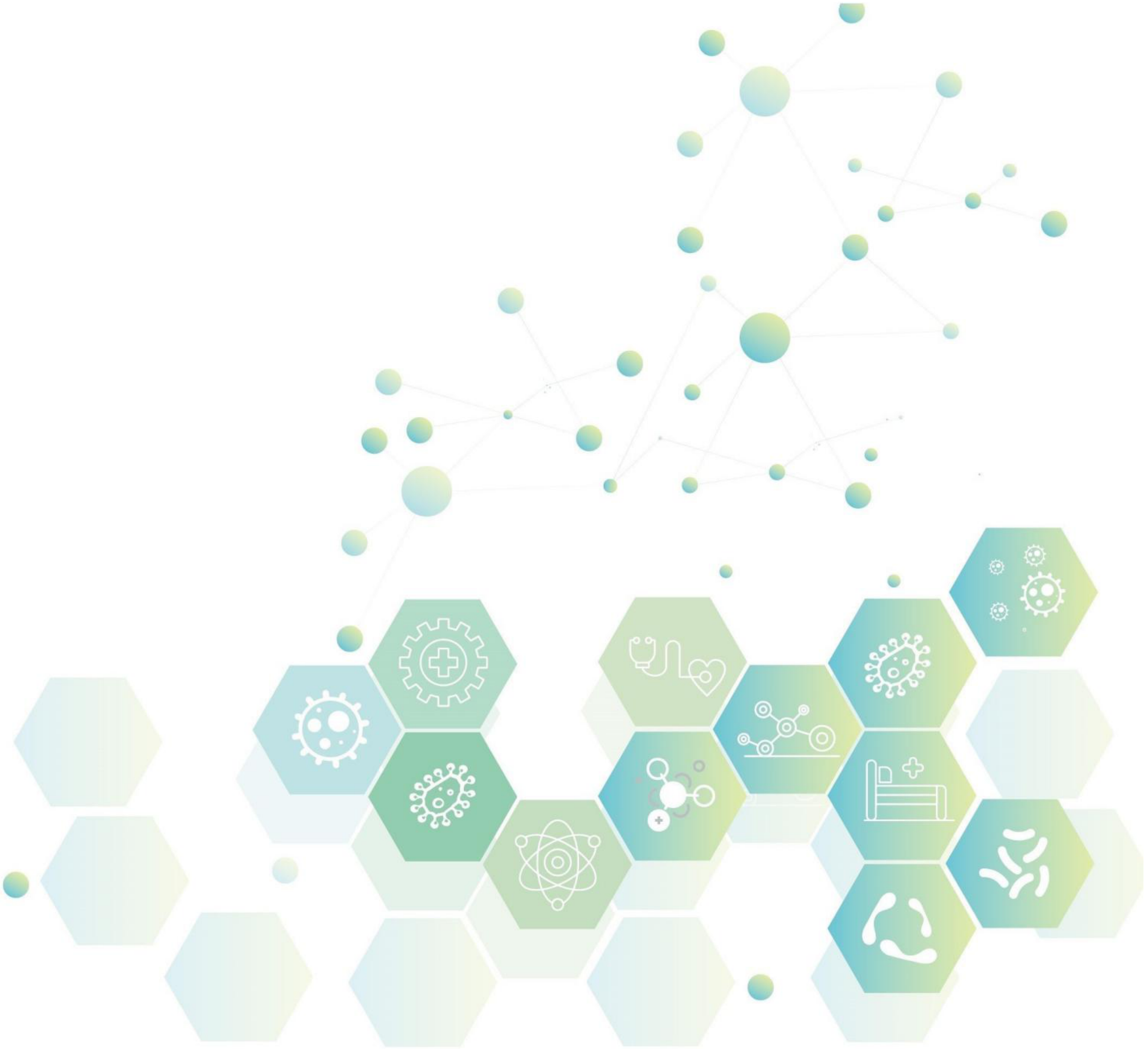

受检者姓名：杨生碧
送检医院：遂宁市中心医院
送检科室：神经内科病区
床号：L01床
样本类型：脑脊液
报告日期：2025-3-2 16:09:05

金域医学
KingMed Diagnostics

SC003LCU60RU6BF

四川金域医学检验中心

SICHUAN KINGMED CENTER FOR CLINICAL LABORATORY

中枢神经系统多种病原体靶向测序报告单

受检者信息

**受检者信息**	姓名：杨生碧	性别：女	年龄：79岁
	住院号/门诊号：25095694		床号：L01床
**样本信息**	样本条码：2365577607	实验编号：CNS25tNGS1135	样本类型：脑脊液
	采样日期：2025-2-28 22:09:33	接收日期：2025-3-1 12:29:48	医院条码/识别码：4183750700
**送检方信息**	送检医院：遂宁市中心医院		
	送检科室：神经内科病区		送检医生：席娅琳
**临床信息**	临床诊断：颅内感染：细菌性脑膜脑炎？ 其他？		
**检测项目信息**	项目名称：中枢神经系统多种病原体靶向测序		检测方法：多重靶向扩增-高通量测序法

病原微生物检测结果

微生物类型	属　名	微生物名称	均一化序列数
**1.特殊病原体列表（分枝杆菌、支原体、衣原体等）**			
未检出			
**2.细菌列表**			
未检出			
**3.真菌列表**			
真菌	念珠菌属<br>*Candida*	白念珠菌<br>*Candida albicans*	57
**4.病毒列表**			
未检出			
**5.寄生虫列表**			
未检出			

均一化序列数：每100K的原始序列中含有该微生物的序列数，均一化序列数越高，则样本含有该微生物的确定性越高。

更多报告服务

耐药基因检测结果

耐药基因分类	耐药基因家族	序列数	建议解析	疑似关联菌
未检出				

疑似关联菌:检出耐药基因不一定是关联病原体携带，也不能说明关联病原体一定会耐药。

可预期耐药信息

病原微生物	可预期耐药信息
未检出	

可预期耐药信息:参考资料《CLSI M100抗菌药物敏感性试验执行标准》

病原微生物解释

病原微生物	相关解释
白念珠菌<br>*Candida albicans*	该菌是真菌，隶属于念珠菌属，可定植在人类和某些恒温动物的消化道或者黏膜，是人类胃肠、皮肤、泌尿生殖道最常见分离的菌种，条件致病菌，机体免疫下降或长时间免疫低下会导致皮肤、黏膜感染、念珠菌血症和其他全身性感染。

检测质控

总序列数	Q30比率	内参检测结果	阳性对照	阴性对照
合格	合格	合格	合格	合格

注:以上检测质控信息说明此次检测测序数据质量合格，结果可信。

*Q30比率:Q30表示错误识别的概率是0.1%，即错误率0.1%，或者正确率是99.9%，Q30比率是指正确率超过99.9%的碱基占比。

检测说明

1.本检测结合多重靶向扩增和高通量测序技术（Targeted NGS,tNGS）对常见病原微生物进行平行检测，通过生物信息技术分析鉴定样本中可疑致病病原微生物，辅助临床医生进行综合分析判断和制定个体化精准治疗方案。
2.本耐药基因检测包含：碳青霉烯类耐药菌（CRO）、耐甲氧西林金黄色葡萄球菌（MRSA）等相关基因检测，微生物的耐药基因型与表型之间存在差异，即使耐药基因检测结果阳性，也不能确认微生物对相应药物一定耐药，本耐药基因检测结果仅供临床医生参考。
3.本检测最低检测限为100copies/mL，低于检测限的病原可能无法检出。
4.本报告仅对本次送检标本负责，无检测人、无审核人签字的报告无效，涂改以及内容缺损无效。
5.本次检出的病原微生物可能与感染有关，病原微生物检测结果列表见附表；建议临床综合患者症状/体征、病史、其他实验室诊断结果等情况进行分析考虑；不作为患者临床诊治或管理的唯一依据，不代表最终诊断结果，分析结果请咨询专科医生。
6.本实验室保留对上述结果的最终解释权，如有疑义，请在报告发布后3个工作日内与我们联系。

金域医学
KingMed Diagnostics

四川金域医学检验中心

SICHUAN KINGMED CENTER FOR CLINICAL LABORATORY

中枢神经系统多种病原体靶向测序报告单

附表：病原体检测结果详细列表

分类	病原微生物	结果	病原微生物	结果	病原微生物	结果
革兰阳性菌	单核细胞增生李斯特菌	(-)	咽峡炎链球菌群*	(-)	粪肠球菌	(-)
	肺炎链球菌	(-)	诺卡菌属*	(-)	屎肠球菌	(-)
	无乳链球菌	(-)	金黄色葡萄球菌	(-)	路邓葡萄球菌	(-)
	停乳链球菌	(-)	结核分枝杆菌复合群	(-)	猪链球菌	(-)
	产气荚膜梭菌	(-)				
革兰阴性菌	脑膜炎奈瑟菌	(-)	按蚊伊丽莎白金菌	(-)	嗜麦芽窄食单胞菌	(-)
	流感嗜血杆菌	(-)	脑膜脓毒伊丽莎白金菌	(-)	铜绿假单胞菌	(-)
	肺炎克雷伯菌	(-)	新洋葱伯克霍尔德菌	(-)	阴沟肠杆菌复合群	(-)
	大肠埃希菌	(-)	洋葱伯克霍尔德菌	(-)	具核梭杆菌	(-)
	鲍曼不动杆菌	(-)	洋葱伯克霍尔德菌复合群	(-)	脆弱拟杆菌	(-)
	布鲁菌属	(-)	鼻疽伯克霍尔德菌	(-)	牙龈卟啉单胞菌	(-)
	坏死梭杆菌	(-)	类鼻疽伯克霍尔德菌	(-)	牙髓卟啉单胞菌	(-)
DNA病毒	单纯疱疹病毒1型(HSV-1)	(-)	巨细胞病毒(CMV)	(-)	人类疱疹病毒8型(HHV-8)	(-)
	单纯疱疹病毒2型(HSV-2)	(-)	人类疱疹病毒6A型(HHV-6A)	(-)	JC多瘤病毒(JCPyV)	(-)
	水痘-带状疱疹病毒(VZV)	(-)	人类疱疹病毒6B型(HHV-6B)	(-)	人类细小病毒B19	(-)
	EB病毒(EBV)	(-)	人类疱疹病毒7型(HHV-7)	(-)	人腺病毒*	(-)
RNA病毒	肠道病毒*	(-)	新型布尼亚病毒	(-)	人嗜T淋巴细胞病毒1型(HTLV-1)	(-)
	甲型流感病毒	(-)	风疹病毒	(-)	人嗜T淋巴细胞病毒2型(HTLV-2)	(-)
	乙型流感病毒	(-)	麻疹病毒	(-)	Pegivirus C	(-)
	副肠孤病毒A型	(-)	日本乙型脑炎病毒	(-)	登革热病毒	(-)
	新型冠状病毒	(-)				
真菌	新型隐球菌	(-)	库德里阿兹威毕赤酵母（克柔念珠菌）	(-)	烟曲霉	(-)
	格特隐球菌	(-)	近平滑念珠菌	(-)	黄曲霉复合群	(-)
	**白念珠菌**	**(+)**	荚膜组织胞浆菌	(-)	土曲霉复合群	(-)
	热带念珠菌	(-)	尖端赛多孢霉	(-)	黑曲霉复合群	(-)
	光滑念珠菌	(-)	粗球孢子菌	(-)		
寄生虫	广州管圆线虫	(-)	刚地弓形虫	(-)	曼氏迭宫绦虫	(-)
	猪带绦虫	(-)	日本血吸虫	(-)	狒狒巴拉姆希阿米巴	(-)
	福勒耐格里阿米巴	(-)				
支原体、立克次体、螺旋体等	人型支原体	(-)	苍白密螺旋体（梅毒螺旋体）	(-)	斑疹伤寒立克次体	(-)
	细小脲原体	(-)	伯氏疏螺旋体	(-)	普氏立克次体	(-)
	恙虫病东方体	(-)	肾脏钩端螺旋体	(-)	肺炎支原体	(-)

注:*标记病原微生物涵盖一种或多种型别(种 、亚型 、血清型等)，其中一种型别或其本身为阳性，则该项病原微生物为阳性，具体涵盖内容如下:

咽峡炎链球菌群:中间链球菌；诺卡菌属:皮疽诺卡菌、圣乔治诺卡菌、巴西诺卡菌、豚鼠耳炎诺卡菌、非洲诺卡菌、德兰士瓦诺卡菌、脓肿诺卡菌、新诺卡菌、星形诺卡菌；人腺病毒:人腺病毒B组、人腺病毒C组、人腺病毒E组；肠道病毒:肠道病毒A组、柯萨奇病毒A6型、柯萨奇病毒A10型、柯萨奇病毒A16型、肠道病毒A71型、肠道病毒B组、埃可病毒、肠道病毒C 组、肠道病毒D组、肠道病毒D68型；

主检人	黄兰兰	审核人	张 雪	批准人	邓方芳

主检实验室:　四川金域　　报告日期: 2025-3-2　16:09:05　　收样点:　遂宁市中心医院-采样点

网址:　www.kingmed.com.cn　　客服电话: 4001-111-120
地址:　四川省成都市成华区龙潭工业园成济路1号

SC003LCU60RU6BF
